# Notes and Formulæ

**Published:** 1889-10

**Authors:** 


### PRACTICAL NOTES AND FORMULÆ.

**Vinum Creosoti Compositum.**—

| | | |
|---|---|---|
| ℞ | Creosoti | ʒij. |
| | Tr. gentianæ | ℥v. |
| | Alcoholis | ʒiv. |
| | Vini. xerici | ad. Oj. M. |

Give two or three tablespoonfuls daily to a phthisical patient when the temperature does not exceed 100° F., and when the bacilli are not yet very numerous.—*L'Union Medicale.*

**Treatment of Headache.**—E. Lloyd James has found that the sipping of cold water relieves headache in a short time, in a large number of cases (*Practitioner*). In acute cases of low pressure headache —*e. g.*, toxic headaches from alcohol and tobacco—exercise and food are potent remedies. Relief is obtained from cardiac stimulants, such as the following:

| | | |
|---|---|---|
| ℞ | Spiritus ammoniæ aromat. | ʒss. |
| | Spiritus chloroformi | Mx. |
| | Aquæ, ad. | ℥j. |

M. Ft. Sol. Sig. Take at a dose.

In more chronic cases prolonged treatment is necessary.—*Ex.*

**Swift's Specific "S. S. S."**—

| | |
|---|---|
| Fluid extract of smilax sarsaparilla | 16 parts. |
| Fluid extract of stillingia sylvatica | 16 parts. |
| Fluid extract of lappa minor | 16 parts. |
| Fluid extract of phytolacca decandra | 16 parts. |
| Tincture of xanthoxylum Carolinianum | 8 parts. |

—*Registered Pharmacist.*

**To Remove Freckles.**—

| | | |
|---|---|---|
| ℞ | Chloride of ammonium | fl. ʒj. |
| | Hydrochloric acid | fl. ʒjss. |
| | Glycerine | fl. ʒj. |
| | Lait virginal (Lait virginal, French cosmetic, prepared by dropping alcoholic tincture of benzine into water until the mixture becomes perfectly white). | fl. ℥jss. |

M. Apply to the freckles morning and evening with a camel's hair brush. Their complete disappearance is said to be speedy.—*Ex.*

**Antiseptic Cotton** may be prepared as follows:

| | |
|---|---|
| Mercury biniodide | p. 8 |
| Potassium iodide | p. 3 |
| Glycerine | p. 120 |
| Distilled water | q. s. ad. p. 2400 |

Dip absorbent cotton in the solution and then dry it.—*Ex.*

**Exalgine** has been quickly adopted in this country and already enjoys considerable popularity. New uses for it, and approved recipes for its administration are being published; here are formulæ for its use in neuralgic dysmenorrhœa, suggested in the *Revue de Ther. Med.-Chir.*:

| | | |
|---|---|---|
| ℞ | Curacao | xii. |
| | Exalgine | ℈ii. |

M. S. A teaspoonful as needed.

| | | |
|---|---|---|
| ℞ | Linden flower water | 100 parts. |
| | Laurel water | 10 parts. |
| | Curacao | 40 parts. |
| | Exalgine | 3 parts. |

M. S. A teaspoonful every half hour until relieved from pain.

**Osmic Acid for Sciatica.**—In a paper read before the Philadelphia Neurological Society, Dr. S. Solis-Cohen reported success in a very obstinate case of sciatica by injecting a one per cent solution of osmic acid into the tissues near the exit of the sciatic nerve. He began by using ten minims, and increased until he finally gave thirty minims at one injection. The injections were made at first daily and later three times a week. The treatment lasted about seven weeks.

[Osmic acid is the tetroxide of osmium. The following is a convenient formula:

| | |
|---|---|
| Osmic acid | 1 part. |
| Glycerine | 40 parts. |
| Distilled water | 60 parts. |

The injections cause severe but temporary pain. They should be continued along the course of the nerve, and not repeated in the same place. At first daily, they should gradually be reduced to once every three days. The fumes of the pure acid are very poisonous and should not be inhaled. The antidote is cautious inhalation of sulphuretted hydrogen.—*Med World.*

**Administration of Cod-liver Oil**-—For the purpose of agreeably modifying both the odor as well as the taste of cod-liver oil, Dr. Seig, in the *Revue de Ther. Med. Chir.*, June 1,, 1889, recommends that it be given in the following formula:

| | | |
|---|---|---|
| ℞ | Cod-liver oil | f ℥ lxx. |
| | Creasote | mxl. |
| | Saccharin | grs. ijss.—M |

The odor of this preparation will resemble that of roast meat.—*Medical News.*

**A Writer** in *Courier Medicale* suggests the following treatment for neuralgia:

| | | |
|---|---|---|
| ℞ | Alcohol. camphorat | p. 90. |
| | Ætheris | p. 30. |
| | Tinct. opii. | p. 6. |
| | Chloroform | p. 20. |

M. Sig. Apply on flannel.

